# Beyond a constant proton relative biological effectiveness: A survey of clinical and research perspectives among proton institutions in Europe and the United States

**DOI:** 10.1002/acm2.14535

**Published:** 2024-11-03

**Authors:** Jakob Ödén, Kjell Eriksson, Suryakant Kaushik, Erik Traneus

**Affiliations:** ^1^ Department of Research RaySearch Laboratories AB Stockholm Sweden

**Keywords:** linear energy transfer, proton therapy, proton track‐ends, relative biological effectiveness, survey

## Abstract

**Purpose:**

Although proton relative biological effectiveness (RBE) depends on factors like linear energy transfer (LET), tissue properties, dose, and biological endpoint, a constant RBE of 1.1 is recommended in clinical practice. This study surveys proton institutions to explore activities using functionalities beyond a constant proton RBE.

**Methods:**

Research versions of RayStation integrate functionalities considering variable proton RBE, LET, proton track‐ends, and dirty dose. A survey of 19 institutions in Europe and the United States, with these functionalities available, investigated clinical adoption and research prospects using a 25‐question online questionnaire.

**Results:**

Of the 16 institutions that responded (84% response rate), 13 were clinically active. These clinical institutions prescribe RBE = 1.1 but also employ planning strategies centered around special beam arrangements to address potentially enhanced RBE effects in serially structured organs at risk (OARs). Clinical plan evaluation encompassed beam angles/spot position (69%), dose‐averaged LET (LET_d_) (46%), and variable RBE distributions (38%). High ratings (discrete scale: 1–5) were reported for the research functionalities using linear LET_d_‐RBE models, LET_d_, track‐end frequency and dirty dose (averages: 4.0–4.8), while LQ‐based phenomenological RBE models dependent on LET_d_ scored lower for optimization (average: 2.2) but congruent for evaluation (average: 4.1). The institutions preferred LET reported as LET_d_ (94%), computed in unit‐density water (56%), for all protons (63%), and lean toward LET_d_‐based phenomenological RBE models for clinical use (> 50%).

**Conclusions:**

Proton institutions recognize RBE variability but adhere to a constant RBE while actively mitigating potential enhancements, particularly in serially structured OARs. Research efforts focus on planning techniques that utilize functionalities beyond a constant RBE, emphasizing standardized LET and RBE calculations to facilitate their adoption in clinical practice and improve clinical data collection. LET_d_ calculated in unit‐density water for all protons as input to adaptable phenomenological RBE models was the most suggested approach, aligning with predominant clinical LET and variable RBE reporting.

## 1 INTRODUCTION

Proton therapy represents a substantial advancement in cancer treatment compared to conventional photon‐based radiation therapy. Leveraging the physical properties of protons, notably the Bragg peak phenomenon, enables precise targeting of tumors while minimizing damage to surrounding healthy tissues. This precision is particularly notable for scanned proton beams, a treatment technique that has achieved broad acceptance internationally. The reduced dose to normal tissues offered by proton therapy holds promise for minimizing side effects and improving treatment outcomes across diverse cancer types and clinical scenarios.

Although ion therapies, including proton therapy, offer these superior physical dose distributions compared to conventional photon‐based radiation therapy, it is also well‐established that the relative biological effectiveness (RBE) for ions varies with depth.^1^ While this variability is more pronounced for heavier ions, radiobiological principles and evidence from both in vitro and in vivo studies suggest a similar trend for protons.^1^, ^2^, ^3^ As the RBE increases with depth, this variability may result in elevated normal tissue toxicities at the distal end of the proton range.^4^, ^5^, ^6^, ^7^, ^8^, ^9^ Despite this, while RBE models accounting for this variability are standard practice for helium and carbon ion therapies, a constant RBE of 1.1 is recommended for proton therapy.^1^ However, this overlooks the multifactorial nature of RBE, which depends on factors such as particle energy, linear energy transfer (LET), tissue characteristics, dose level, dose rate, and the specific biological endpoint under consideration. Although the clinical evidence for variable proton RBE currently is considered statistically weak,^1^, ^9^ this oversight in proton therapy may still potentially lead to suboptimal treatment outcomes in terms of both tumor control probability (TCP) and normal tissue complication probabilities (NTCP). However, even with limited clinical evidence, RBE‐related quantities like dose‐averaged LET (LET_d_), proton track‐end frequency, or “dirty dose” have been suggested to be minimized in critical structures to mitigate a potential enhanced RBE to further improve the therapeutic ratio using proton therapy.^10^

To investigate the clinical use and future demands concerning clinical application of variable proton RBE, a recent study from the European Particle Therapy Network (EPTN) work package 6 (WP6) conducted a survey among all European proton institutions.^11^ The findings revealed that none of the institutions employed variable RBE models for clinical dose prescriptions, although each institution actively mitigated variable RBE effects in organs at risk (OARs) through diverse special planning strategies. The study identified some key requirements, emphasizing the necessity for standardized calculation tools for LET and variable RBE to facilitate the collection of enhanced clinical data on variable RBE effects. These demands align with insights from recent reviews on LET reporting^12^ and on clinical studies using variable proton RBE.^9^ Notably, various research findings have utilized LET^10^, ^13^, ^14^, ^15^ and variable RBE models^16^, ^17^, ^18^, ^19^, ^20^, ^21^, ^22^, ^23^ for proton therapy, alongside additional functionalities beyond constant proton RBE, such as the proton track‐end frequency^24^, ^25^ and dirty dose concepts.^26^, ^27^ Recently, both LET_d_ optimization and evaluation have also become clinically accessible in commercial treatment planning systems (TPSs), with the latter enabling plan evaluation using variable RBE models based on physical dose and LET_d_ distribution as input parameters.^28^

Given the substantial interest in planning and evaluating proton treatments beyond the constant RBE, this study seeks to offer an updated, comprehensive overview of current clinical practices, ongoing research initiatives, and the potential for integrating functionalities beyond the constant RBE in clinical practice. This will be achieved through a survey conducted among proton therapy institutions in both Europe and the United States.

## 2 METHODS

### 2.1 Functionalities beyond a constant proton RBE

**FIGURE 1:**
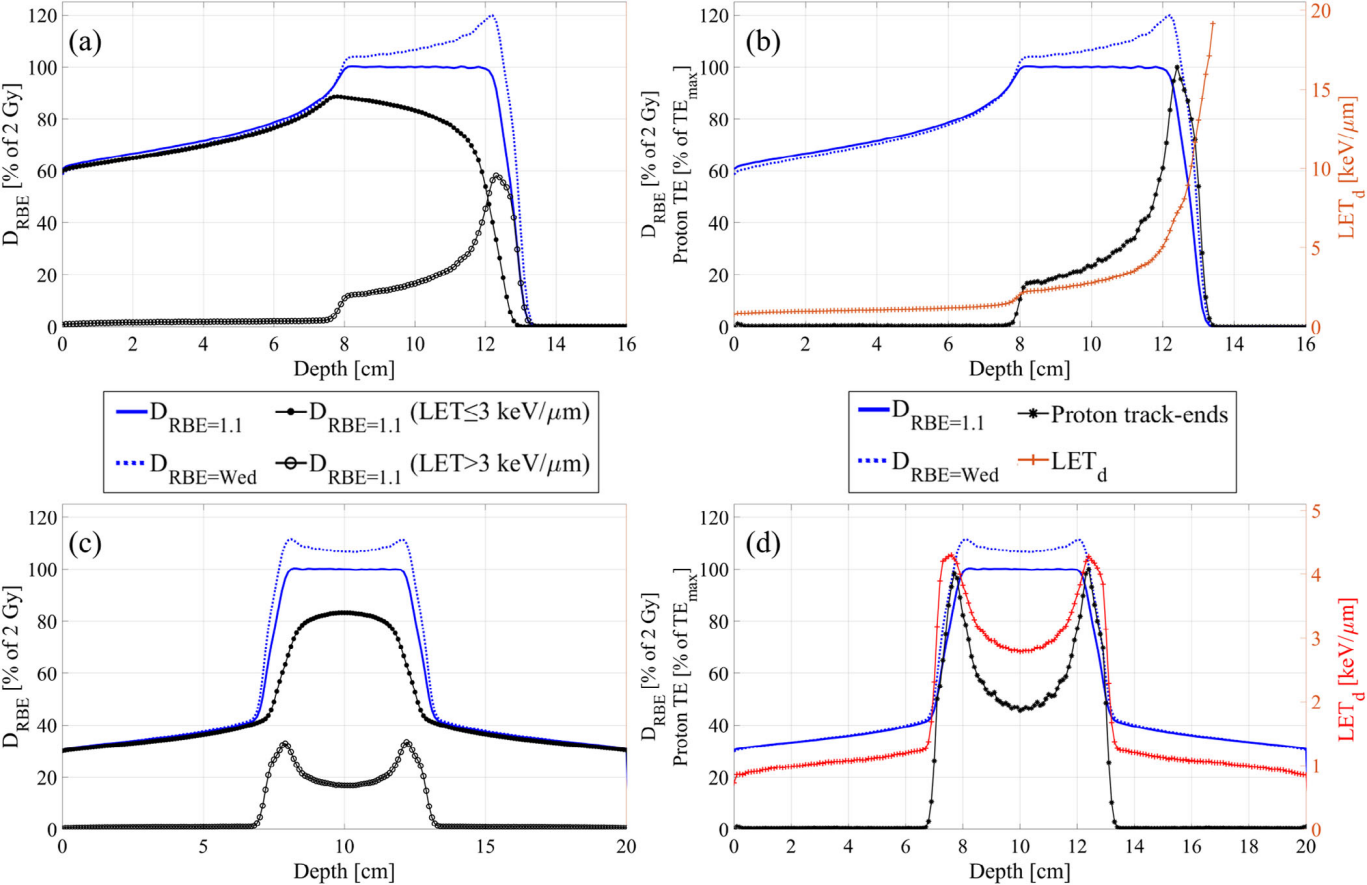
Central axis depth profiles in water for 4 cm wide SOBPs. (a) Single field SOBP with RBE‐weighted dose using RBE = 1.1 (D_RBE = 1.1_), RBE‐weighted dose with the Wedenberg RBE model^37^ with α/β = 3 Gy (D_RBE = Wedenberg_), clean dose (D_RBE = 1.1_ deposit by protons with LET ≤ 3 keV/µm) and the dirty dose (D_RBE = 1.1_ deposit by protons with LET > 3 keV/µm). (b) D_RBE = 1.1_ and D_RBE = Wedenberg_ with the proton track‐end (TE) distribution for primary protons (left *y*‐axis) and LET_d_ considering all protons for D_RBE = 1.1_ > 1% (right *y*‐axis). [(c) and (d)] Same as (a) and (b), respectively, but for two parallel opposing proton SOBPs. All calculations were done in a research version of RayStation v11B. LET, linear energy transfer; LET_d_, dose‐averaged LET; RBE, relative biological effectiveness; SOBP, spread‐out Bragg peaks.

In this study, functionalities beyond a constant proton RBE refers to using variable proton RBE models, as well as RBE‐related functionalities using LET, proton track‐end frequency, and the dirty dose concept. The LET is commonly quantified through track‐ or dose‐averaging, where the track‐average LET (LET_t_) computes the mean LET of individual particles within a field, while the LET_d_ assigns weight to each particle's LET based on the energy it deposits in the volume of interest. This weighting acknowledges that high‐LET particles deposit more dose and thus contribute more significantly to observed biological effects.^29^ Although LET_t_ and LET_d_ both correlate with RBE and can be useful for plan optimization and evaluation as indicators of elevated RBE, the LET_d_ is adopted as the primary LET metric throughout this study, consistent with recent literature.^10^, ^12^, ^13^, ^30^, ^31^ However, the LET_t_ was also available for plan evaluation in research setting and the specific preferences regarding LET reporting were also addressed through a survey question (see question 20 in Supplementary Material). Figure 1 shows the most relevant quantities in this study for proton spread‐out Bragg peaks (SOBPs) using one or two parallel opposing fields. The spatial correlation between the increased RBE‐weighted dose (using a variable RBE model) and LET_d_, proton track‐ends, and the dirty dose is evident. This correlation highlights that LET_d_, proton track‐end frequency, and dirty dose serve as suitable proxies for elevated proton RBE in the optimization and evaluation of proton therapy plans, as demonstrated in prior studies.^17^, ^24^, ^25^, ^26^

#### 2.1.1 Variable proton RBE models

The proton RBE is defined as the ratio of the absorbed dose of reference radiation (most commonly low LET MV photons) to the absorbed dose of protons that produces the same biological effect. Typically, a constant RBE of 1.1 is used regardless of the clinical endpoint and underlying biology, implying that proton doses are, on average, about 10% more biologically damaging than MV photons. However, spatial variations in proton RBE as indicated by Figure 1 could potentially increase NTCP, especially at the end of the proton range, or potentially reduce the TCP in certain cases. To cope with this, multiple mathematical RBE models have been proposed for usage in proton therapy treatment planning. Schematically one can divide them into phenomenological models and mechanistic‐inspired models. In this study, the focus is on two specific types of nonclinical phenomenological variable RBE models dependent on the LET_d_. First, the linear LET_d_‐RBE model where the RBE is a linear function of LET_d_ as,

(1) $$\mathrm{RBE}=a+b\cdot {\mathrm{LET}}_{d},$$

where *a* and *b* are empirical fitting parameters determining the intercept (i.e., RBE value for LET_d_ = 0 keV/µm) and slope of the model, respectively. Values of *a* ≈ 0.95–1.0 and *b* ≈ 0.04–0.09 µm/keV have been reported in the literature.^9^, ^31^, ^32^, ^33^ Note that the constant RBE = 1.1 and the dose‐LET_d_ product^31^ are special cases of Equation (1) by setting *a *= 1.1 and *b *= 0 µm/keV, or *a *= 0 and *b *= 1 µm/keV, respectively.

The second considered phenomenological RBE model type was the LET_d_‐dependent RBE model based on the linear‐quadratic (LQ) dose‐response (LQ‐LET_d_‐RBE model). Comprehensive summaries of such LQ‐LET_d_‐RBE phenomenological RBE models can be found elsewhere.^34^, ^35^ In this study, the LQ‐LET_d_‐RBE models were defined as,

(2) $$\mathrm{RBE}=\frac{1}{2d_{p}}\left[\sqrt{{\left(\frac{\alpha }{\beta }\right)}_{x}^{2}+4d_{p}{\left(\frac{\alpha }{\beta }\right)}_{x}{\mathrm{RBE}}_{\max}+4d_{p}^{2}{\mathrm{RBE}}_{\min}^{2}}-{\left(\frac{\alpha }{\beta }\right)}_{x}\right],$$

where *d*_p_ is the proton fraction dose, (*α*/*β*)_*x*_ is the ratio of the photon tissue‐specific LQ parameters, and *RBE*_*max*_ and *RBE*_*min*_ are defined here as,

(3) $${\mathrm{RBE}}_{\max}=p_{0}+\frac{p_{1}}{{\left(\alpha /\beta \right)}_{x}}{\mathrm{LET}}_{d},$$

(4) $${\mathrm{RBE}}_{\min}=p_{2}+p_{3}f\left({\mathrm{LET}}_{d}\right),$$

where *p_0_*, *p_1_*, *p_2_* and *p_3_* are fitting parameters and *f*(LET_d_) is a model specific LET_d_‐dependent function.

The parameter values and the specific function *f*(LET_d_) for the three LQ‐LET_d_‐RBE models specifically used in this study (Carabe et al.,^36^ McNamara et al.^3^ and Wedenberg et al.^37^) are summarized elsewhere.^35^ Note that both the linear LET_d_‐RBE and LQ‐LET_d_‐RBE models can be used clinically for plan evaluation in the RayStation TPS (RaySearch Laboratories AB, Stockholm, Sweden). This is accomplished using the Python scripting interface, where users can enter their specific RBE models and model parameters, utilizing the physical dose and LET_d_ distributions as inputs.^28^ For optimization, these variable RBE models are; however, strictly restricted to specific research versions of RayStation.

In addition to the two types of phenomenological RBE models dependent on LET_d_, we also present the option of mechanistic‐inspired RBE models (see questions 3 and 21 in Supplementary Material), although no such implementations are currently available for proton therapy treatment planning. Specifically, the microdosimetric kinetic model (MKM), local effect model (LEM), double‐strand break (DSB) model, and repair‐misrepair‐fixation (RMF) model are stated as options. Unlike the phenomenological models considered here, which describe an empirical relationship between LET_d_ and RBE, mechanistic‐inspired models aim to predict biological damage by considering both the physical and chemical interactions of radiation within the cell (e.g., DNA DSB) and the subsequent biological processes (e.g., cell repair) that lead to lethal cell damage. Comprehensive summaries and comparisons of such mechanistic‐inspired RBE models are available in the literature.^35^, ^38^

#### 2.1.2 RBE‐related functionalities

Nonclinical RBE‐related functionalities using LET, proton track‐ends, and the dirty dose implemented in research versions of RayStation were considered in this study. LET here refers to either LET_d_ or LET_t_ in a voxel if the plan dose exceeds a user‐specified dose threshold in that voxel. If the dose is below the threshold, the averaged LET in such voxel is not considered during optimization or evaluation.^28^ This ensures only clinically relevant voxels are included since LET in a low dose voxel is of minor clinical interest.^12^ The proton track‐ends frequency refer to the number of primary protons stopping in each voxel,^24^ and the dirty dose concept refers to that the dose distribution is divided into two parts: the clean and the dirty dose. The clean dose and dirty dose are deposited by protons with a LET below and above a user specified LET threshold, respectively.^17^, ^26^ Based on initial findings, an LET threshold in the range of approximately 2 to 7 keV/µm is deemed appropriate for OARs in proton therapy.^26^ The dirty dose optimization function utilized the standard one‐sided quadratic penalty on voxel dirty dose deviations from a desired dirty dose level. The LET_d_ and proton track‐end optimization functions were implemented as the standard optimization functions for minimum and maximum voxel doses, with a one‐sided quadratic penalty on voxel deviations from a desired level of LET_d_ or proton track‐end frequency.^24^, ^28^ Hence, given the correlations of these quantities with RBE observed in Figure 1, optimization functions that penalize high values of LET_d_, proton track‐ends, or dirty dose in OARs can reduce RBE in these regions, likely leading to a decrease in NTCP. Conversely, penalizing low values of these metrics in the tumor volume can increase RBE and, consequently, may improve the TCP.

### 2.2 Survey

To gain insights into the clinical adoption, cutting‐edge research endeavors, and prospects related to functionalities beyond the constant proton RBE, a comprehensive survey was designed and distributed in May 2023, with a deadline for responses set for September 2023.

#### 2.2.1 Selection of proton institutions

The survey primarily revolved around the utilization of functionalities beyond the application of a constant proton RBE in both clinical and research environments, with particular emphasis on the use and potential of research functionalities on variable RBE models, LET, proton track‐ends and the dirty dose concept available in RayStation. Thus, to provide insights into both clinical and research perspectives, participating proton institutions were required to have access to and actively use these functionalities in research and/or clinical practice. With these criteria in mind, 19 institutions—ten in Europe and nine in the United States—were identified and contacted to complete the questionnaire via a provided link to the online survey.

#### 2.2.2 Survey design

The survey was structured as an online questionnaire tailored for compatibility with personal computers, tablets, and smartphones, utilizing the web platform Netigate (Netigate AB, Stockholm, Sweden). The questionnaire commenced with general inquiries regarding the respondent's profession, years of experience, and specific research versions utilized. This was followed by the main section focusing on the utilization of functionalities beyond a constant proton RBE. This main segment of the online questionnaire comprised 25 questions in total, categorized into clinical use, research and experimental activities, and future applications. Although there was an approximate uniformity in the distribution of questions across each subcategory, the survey design exhibited a deliberate emphasis on inquiries concerning research activities and anticipated future applications. Multiple‐choice responses were predominant, with the option for respondents to provide free‐text comments. The estimated completion time for the survey was approximately 30 min, with responses saved continuously to enable respondents to pause and resume at their convenience. The full questionnaire can be accessed in the Supplementary Material.

## 3 RESULTS

Nine out of ten European institutions and seven out of nine institutions in the United States completed the survey, giving a response rate of 84% (16/19 institutions). While all participating institutions agreed to share the resulting data, a majority (75%) requested anonymity. Consequently, all results here are presented anonymously. Among the 16 surveyed institutions, three institutions exclusively indicated research purposes. Hence, the percentage concerning current clinical use (Section 3.1) relates to the subset of 13 institutions currently engaged in clinical activities. Except for that section, unless otherwise specified, percentages within parentheses about a question refer to the 16 complete responses. Most respondents identified themselves as physicists (81%), followed by researchers (13%) and doctoral candidates (6%). RayStation experience levels were approximately evenly distributed among the four options: 1–3 years (19%), 4–6 years (31%), 7–9 years (31%), and 10+ years (19%).

### 3.1 Current clinical use

**FIGURE 2:**
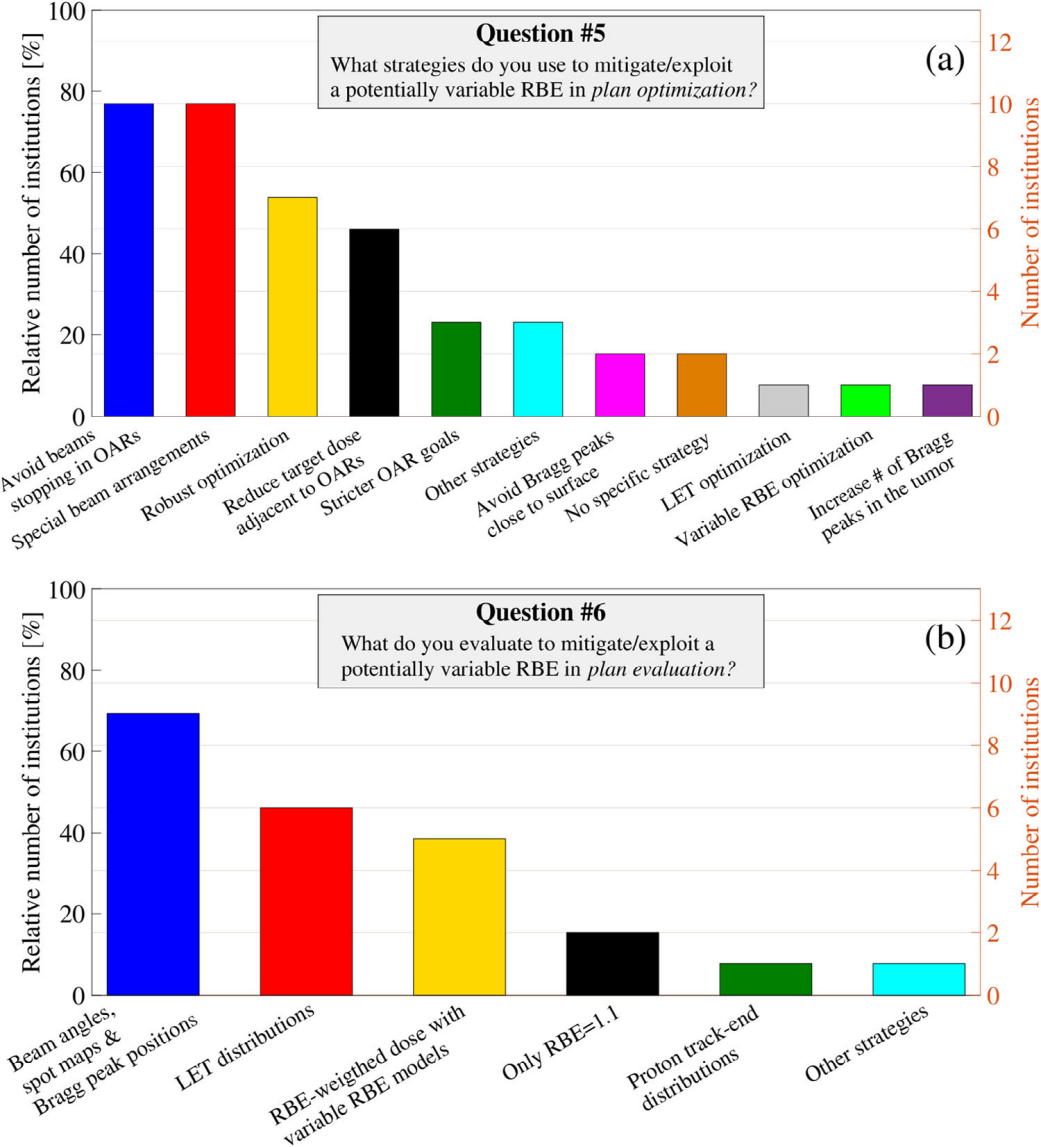
Responses to survey questions (a) number 5 and (a) 6 are found in the Supplementary Material. Note that these were multiple‐choice questions and were reported for the subset of 13 institutions currently engaged in clinical activities.

A diverse range of treatment sites were treated across the participating 13 clinical institutions. Notably, head & neck (H&N) (100%), intracranial lesions (100%), gastrointestinal cancers (85%), cranio‐spinal irradiation (77%), breast cancer (77%), lung cancer (69%), lymphomas (62%), sarcomas (54%), urogenital cancers (54%), and liver cancer (54%) were treated in at least half of the clinically active institutions. Most of them (85%) actively addressed the potential variability in RBE in clinical practice. While a subset of institutions (15%) aimed to utilize the variability of RBE in the tumor for clinical purposes, the emphasis primarily centered on sequentially structured OARs. Specifically, OARs in H&N and intracranial regions were prioritized, including critical structures such as the brainstem, chiasm, cochlea, optic structures, and spinal cord. This reflects the prevalent treatment of targets in these regions across all participating institutions. In clinical proton plan optimization, efforts to mitigate potential enhanced RBE primarily focused on avoiding beam stopping in OARs (77%) and employing special beam arrangements (77%). However, several other strategies were also utilized, as illustrated in Figure 2a. Clinical plan evaluations beyond a constant RBE focused on assessing beam angles, spot maps, and Bragg peak positions (69%), as well as evaluating LET_d_ distributions (46%), as illustrated in Figure 2b. Additionally, some institutions (38%) reported the use of variable proton RBE models whereas only a minority (13%) perform no evaluations beyond RBE = 1.1. The variable RBE evaluation was primarily achieved through scripting capabilities, employing various implementations of linear LET_d_‐RBE (3/5 institutions) and/or LQ‐LET_d_‐RBE models (4/5 institutions).

### 3.2 Research and experimental activities

All 16 participating institutions had explored the research functionalities beyond RBE = 1.1, primarily focusing on serially structured OARs (100%), although use in parallel structured OARs was also reported (38%). The interest in exploiting variable RBE in tumors was also higher for research purposes (approximately 25%) compared to clinical use (13%). The focus was generally on primary treatment courses, although a subset of the institutions (25%) also reported research associated with variable RBE in re‐irradiation cases.

The usage and ratings of the research functionalities beyond a constant RBE are summarized in Table 1. As seen, the LET_d_ function was predominantly explored both in optimization and evaluation (81%, 13/16 institutions), whereas the other functions were explored by 38%–63% of the institutions. Noticeable, the LET_d_, linear LET_d_‐RBE, dirty dose, and proton track‐end functions received high and roughly equivalent ratings (discrete scale: 1–5) for both optimization and evaluation (averages: 4.0–4.8), whereas the LQ‐LET_d_‐RBE function scored substantially lower for optimization (average: 2.2) compared to evaluation (average: 4.1). The LET_t_ was only available for evaluation and scoring similar as LET_d_ although fewer institutions had used it. Note also that the spread in ratings was slightly larger for evaluation compared to optimization for all functions.

**TABLE 1:** The number of institutions that have explored research functionalities beyond a constant RBE, together with their ratings based on the responses to survey questions number 7–12 found in the Supplementary Material.

	Number of institutions		Average rating [min, max]	
Research functionality	Optimization	Evaluation	Optimization	Evaluation
Dirty dose with LET threshold	10 (8)	9 (7)	4.4 (4, 5)	4.0 (2, 5)
LET_d_ with dose threshold	13 (11)	13 (12)	4.6 (4, 5)	4.3 (3, 5)
LET_t_ with dose threshold	–	7 (5)	–	4.2 [2, 5]
Linear LET_d_‐RBE model	6 (5)	9 (8)	4.8 (4, 5)	4.1 (2, 5)
LQ‐LET_d_‐RBE model	6 (5)	9 (8)	2.2 (1, 4)	4.1 (1, 5)
Proton track‐ends	9 (8)	7 (7)	4.1 (4, 5)	4.3 (3, 5)

*Note*: The table includes the number of institutions that have used each functionality, the number of these institutions that have rated the functionality's usefulness (in parentheses), and the average, minimum, and maximum ratings of usefulness (on a discrete scale from 1 to 5, with 5 being the most useful). Note that the LET_t_ with dose threshold functionality was only implemented for plan evaluation, whereas all other functionalities were implemented for both optimization and evaluation.

Abbreviations: LET, linear energy transfer; LET_d_, dose‐average LET; LET_t_, track‐average LET; LQ, linear‐quadratic; RBE, relative biological effectiveness.

### 3.3 Future applications

**FIGURE 3:**
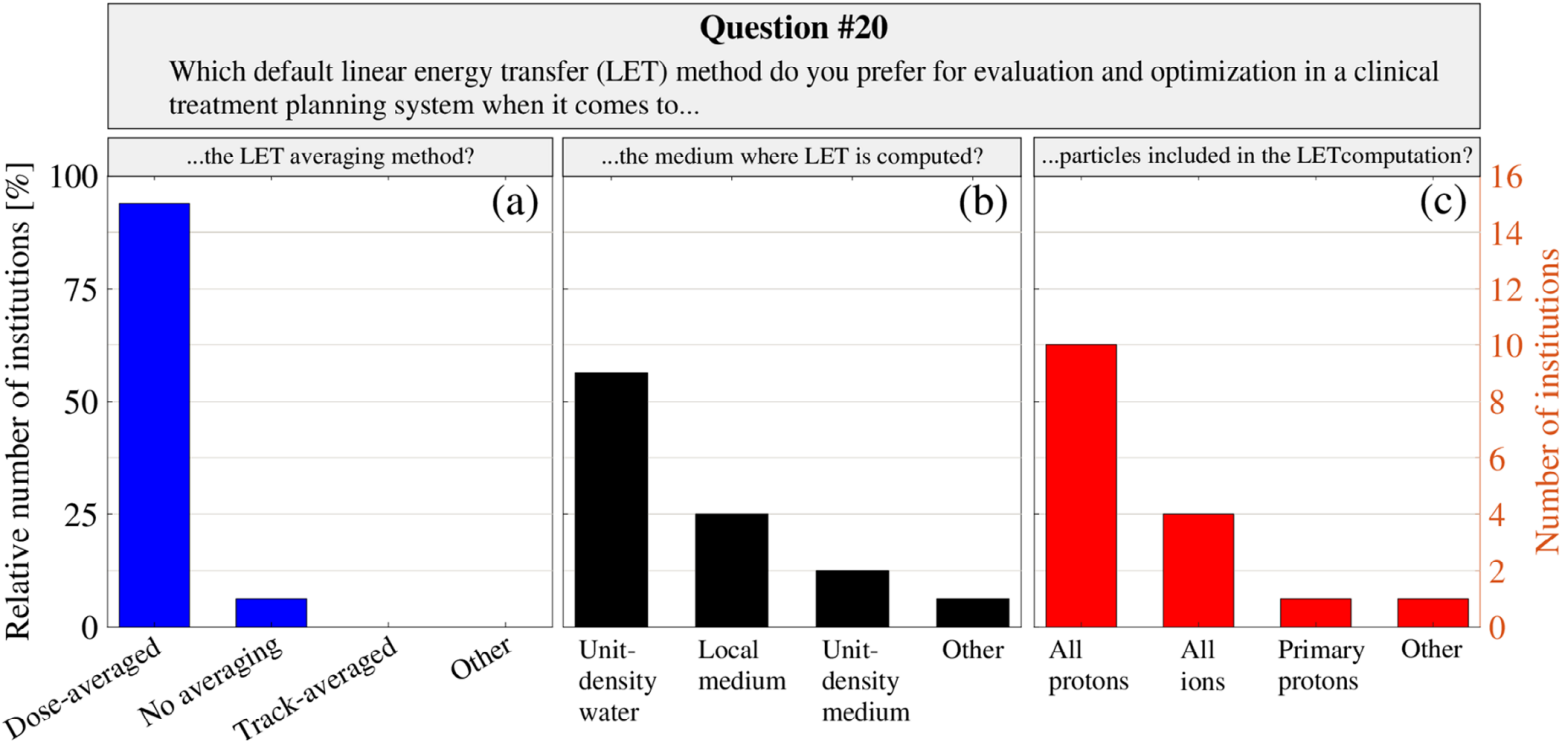
Responses to survey question number 20 are found in the Supplementary Material. Note that all three parts (a, b, and c) of the question were single‐answer questions and reported for all the 16 surveyed institutions.

**FIGURE 4:**
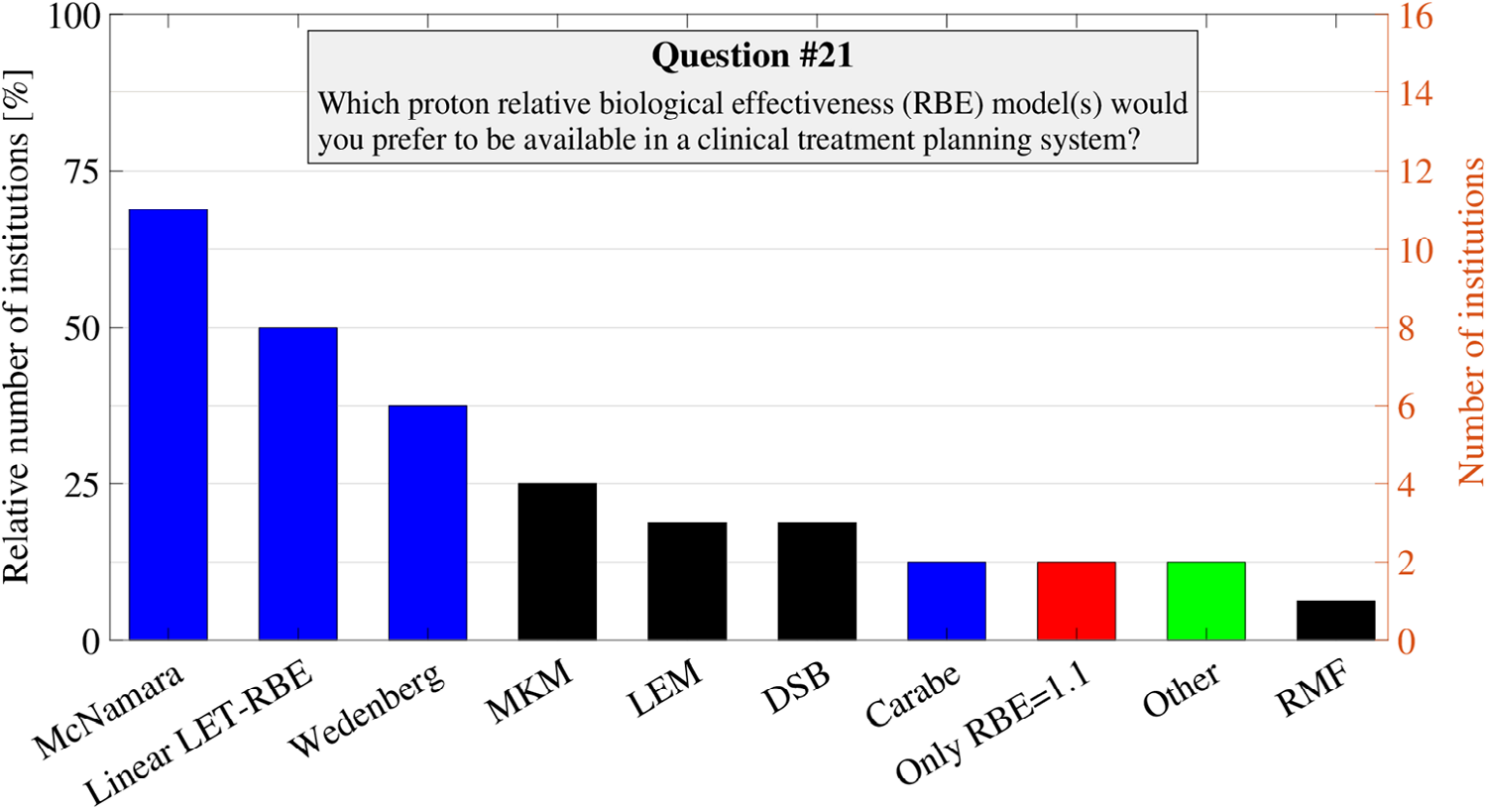
Responses to survey question number 21 are found in the Supplementary Material. Phenomenological RBE models dependent on the LET are marked with blue bars (linear LET‐RBE model, Carabe et al.,^36^ McNamara et al.^3^ and Wedenberg et al.^37^), mechanistic‐inspired variable RBE models are marked with black bars MKM, LEM, RMF model, and DSB model), the constant RBE of 1.1 with a red bar, and other models are group with a green bar. Note that this was a multiple‐choice question and reported for all the 16 surveyed institutions. DSB, double‐strand‐break; LET, linear energy transfer; LEM, local effect model; MKM, microdosimetric kinetic model; RBE, relative biological effectiveness; RMF, repair‐misrepair‐fixation.

The preferred LET reporting for future clinical applications is summarized Figure 3. All institutions but one favored reporting LET as LET_d_ (94%), whereas there were some discrepancies regarding the medium and particles to include for the LET computation. Nonetheless, the majority preferred LET to be computed in unit‐density water (56%), for all protons (63%). All institutions intend to utilize functionalities beyond a constant RBE clinically when available, focusing on plan evaluation rather than optimization. The majority (63%) believe that the functionalities will be used in specific cases for evaluation, while the remaining institutions (38%) anticipate routine use for every patient when clinically available. For optimization, no institution reported anticipated routine utilization of functionalities beyond a constant RBE in the near future. However, a majority (69%) aimed to employ them in specific cases when available in a clinical optimization setting. Aligned with current clinical practices, most institutions favored phenomenological RBE models dependent on LET_d_ over mechanistic‐inspired RBE models for future integration into clinical TPSs, as shown in Figure 4. Notably, a subset of respondents emphasized the necessity for flexible parameter definition of such LET‐dependent models, as outlined in Equations (1) and (2).

## 4 DISCUSSION

In vitro studies have consistently shown an elevated RBE at the distal end of a proton SOBP.^1^, ^3^ Despite these observations, the widespread clinical adoption of a constant proton RBE of 1.1 persists globally,^1^ although the substantial response rates (84% in this study and 100% in the EPTN WP6 survey^11^) underscore the interest in variable proton RBE. This distinction is accentuated by comparing it to the average online survey response rate of 44.1% from a comprehensive meta‐analysis.^39^ This interest in variable proton RBE, especially concerning serially structured OARs, can likely be attributed to the reports on potential normal tissue toxicities resulting from elevated RBE.^4^, ^5^, ^6^, ^7^, ^8^, ^9^ Nevertheless, none of the surveyed institutions reported employing dose prescription using variable RBE models in clinical practice, consistent with the EPTN WP6 survey. However, five institutions indicated the utilization of variable RBE models for clinical plan evaluation in this study (see Figure 2b). This was facilitated by the availability of clinical LET_d_ evaluation, coupled with scripting capabilities, enabling flexible assessments of linear LET_d_‐RBE and LQ‐LET_d_‐RBE models in clinical plan evaluation.^28^ Beyond this, all institutions reported mitigating the potentially variable RBE in OARs using similar planning methods as in the EPTN WP6 survey (see Figure 2a). Furthermore, all surveyed institutions in this study expressed the belief that at least a subset of proton therapy patients could potentially benefit from the future implementation of variable RBE models and aim to use them for at least plan evaluation when becoming clinically available. A minority of the institutions (19%) even believe in using variable RBE for NTCP evaluations in plan comparisons for the selection of patients, as explored in recent research,^17^, ^18^ ^20^, ^22^, ^26^ whereas no institution plans to use variable RBE in TCP calculations.

In alignment with the recent LET harmonization efforts in Europe,^13^ the majority in this study preferred reporting of LET as LET_d_, computed in unit‐density water for all protons. Nonetheless, discrepancies aligning with a recent systematic review on LET reporting^12^ were observed regarding the handling of LET from the secondary particle spectra and the choice of scoring medium (see Figure 3). While these findings highlight the critical need for standardizing LET reporting within the international proton therapy community, it is noteworthy that the preferred reporting method in this study coincides with the clinical LET reporting utilized in RayStation and the primary LET metric in this study. These consistencies suggest that the survey results may be biased by the specific characteristics of the TPS employed. Therefore, caution should be exercised in generalizing the findings to institutions using other systems. Nevertheless, the results of this study still advance efforts toward standardizing LET reporting, proposing the standardization of LET_d_ computed in unit‐density water for all protons. Such standardization would foster consistency and comparability across different proton therapy institutions, echoing previous research emphasizing this need.^9^, ^12^, ^13^

The assessment of research functionalities beyond a constant proton RBE generally yielded high ratings for both optimization and evaluation, averaging between 4 and 5 on a scale of 1–5 (see Table 1). Although these average ratings are based on relatively few votes (5–12 votes per function), this indicates the promising potential for utilizing variable RBE models as well as other RBE‐related functions in proton therapy planning, which is already emphasized in several publications.^1^, ^9^, ^10^, ^17^, ^25^, ^26^ This is further supported by the free‐text comments from several respondents that LET, proton track‐end, dirty dose, and RBE distributions were highly beneficial, particularly when utilized in conjunction with complex multi‐field optimization. In such cases, understanding the resulting distributions may pose challenges, making 3D distributions with corresponding volume histograms of these metrics particularly valuable for treatment planning and evaluation. This becomes particularly crucial when these functionalities are applied in demanding re‐irradiation scenarios, as reported by certain institutions, where it is preferable to couple them with optimization functions specifically tailored for re‐irradiation cases using effective doses.^40^ Notably, even evaluation of LET_t_ scored high (average: 4.2) suggesting that LET evaluation may be valuable regardless of the averaging method, as similar trends persist even though the exact magnitudes may differ.

The rating for LQ‐LET_d_‐RBE models for optimization was notably lower compared to the other functions, which is attributed to observations that it seems to primarily reduce dose rather than LET_d_, which some respondents sought. In contrast, the evaluation score for LQ‐LET_d_‐RBE models was comparable to other functions (see Table 1). However, despite this, only few clinics reported interest in more mechanistic‐inspired RBE models (like MKM, LEM, DSB, and the RMF model)^38^ beyond flexible implementations of the linear LET‐RBE and LQ‐LET‐RBE models for future clinical applications (see Figure 4). Even though the exact formulation of the LQ‐LET‐RBE models varies, the results from a recent study highlights the importance of incorporation an inverse dependence on (*α*/*β*)_x_, as in Equation (3), to perform better across the available data.^34^ While such phenomenological RBE models dependent on LET_d_ may appear to be the most intuitive approach for future planning of proton therapy plans, it is also important to acknowledge the inherent uncertainty associated with the available models.^1^, ^9^ The complexity of biological responses to radiation, as well as the variability in RBE across different tissues and biological endpoints, can introduce considerable uncertainty in predicting RBE values accurately.^16^, ^34^ While recent initiatives have addressed certain uncertainties, the issue of radiobiological effect persists due to insufficient clinical data for relevant endpoints.^9^ Consequently, it could be argued that full utilization of variable RBE optimization may presently be unsuitable for clinical treatment planning using the available models.^1^, ^10^, ^34^, ^41^ This is further supported by the cautious responses in this survey regarding the future application of RBE optimization compared to its use for plan evaluation, and also aligns with the desire expressed by several institutions that these RBE models should be implemented with some flexibility to enable exploration and modification during proton planning and evaluation. Considering these uncertainties in the RBE models, toxicity mitigation strategies substituting RBE such as optimization on LET_d_, proton track‐end frequency, and the dirty dose offer practical alternatives for treatment planning.^1^, ^10^, ^17^ Notably, all these strategies correlate spatially with potentially elevated RBE (see Figure 1). By leveraging such physical functions for OARs, clinicians can potentially mitigate the uncertainties associated with variable RBE models and optimize treatment plans with greater confidence. Notably, since LET_d_ optimization is most used^10^ and has recently become clinically available,^28^ it is likely to anticipate further utilization of this specific RBE‐related function in clinical plan optimization moving forward. Another viable planning approach could be to maintain the target dose with RBE = 1.1, while concurrently addressing the potential impact of variable RBE in OARs through the utilization of a secondary variable RBE model in those regions.^17^, ^20^, ^21^ Such strategy might also be combined with any additional RBE‐related functionality presented in this study.

Although this study aims to report generalizable findings, it should be noted that the study is limited by the criterion that selected institutions all had RayStation TPS with research functionalities beyond a constant RBE available. This criterion may introduce selection bias and somewhat limit the generalizability of findings to institutions utilizing other TPSs. However, it is worth noting that among the approximately 180 proton institutions worldwide in operation, under construction, or in the planning stage,^42^ over 120 have selected RayStation.^28^ Moreover, it was also reported as the most prevalent system for LET and RBE calculations in the comprehensive EPTN WP6 survey.^11^ This suggests that the 16 participating institutions in this study are likely to be representative of the broader proton therapy community, particularly regarding LET and RBE calculations, which were the focus of this study. Additionally, a couple of the surveyed institutions clinically employ a TPS from a different vendor, while exclusively utilizing the research functionalities of RayStation. This usage aligns with a recent European multicentric study on the harmonization of clinical LET reporting in proton radiotherapy, where various research Monte Carlo simulation tools such as FRoG, GATE, TOPAS and TRiP98 were used alongside RayStation for the LET calculations.^13^ Moreover, the primary findings regarding the utilization of planning and evaluation beyond a constant proton RBE generally align with recent studies exploring the application of proton LET and RBE.^9^, ^10^, ^11^, ^12^, ^13^, ^21^ Therefore, while acknowledging this limitation, the study's findings still offer valuable insights into current practices and prospects beyond a constant RBE within the proton therapy community.

## 5 CONCLUSIONS

This comprehensive survey has provided detailed insights into current clinical practices, ongoing research, and future applications related to RBE in proton therapy institutions across Europe and the United States. Notably, all surveyed institutions are actively addressing elevated RBE in OARs, while adhering to current guidelines that recommend a constant RBE for prescription and reporting. RBE mitigation strategies primarily involve specialized beam arrangements and, where available, evaluations of LET_d_ and variable RBE. Research efforts are increasingly focused on developing planning techniques that go beyond a constant RBE using various RBE‐related functions including LET_d_, proton track‐end frequency, and the dirty dose concept. To advance the understanding of proton RBE, gather more robust clinical data, and address RBE‐related challenges, it is crucial to encourage institutions to adopt standardized LET and RBE evaluations in clinical practice. According to the survey results, the most suggested approach is to use LET_d_ calculated in unit‐density water for all protons as input to adaptable phenomenological RBE models. This approach aligns with the predominant clinical LET and variable RBE reporting. Following this, it would be logical to explore clinical optimization options aimed at minimizing adverse effects using appropriate variable RBE‐related functionalities, while ensuring adherence to the RBE = 1.1 requirement in the tumor.

## AUTHOR CONTRIBUTIONS

Dr. Jakob Ödén (corresponding author) led the study design, contributed to the technical development of the software/tools, and managed data collection and analysis. Dr. Jakob Ödén wrote the manuscript, handled all revisions, and incorporated feedback from co‐authors, conducting the final review before submission. Kjell Eriksson played a significant role in the study design and provided critical input during the manuscript's review process, offering feedback and suggestions to refine the content during the final manuscript review and revision stages. Suryakant Kaushik was involved in the study design and played a significant role in the manuscript's critical review, providing substantial feedback and suggestions during the final manuscript review and revision stages. Dr. Erik Traneus served as the technical lead for the software/tool development, overseeing its design and implementation. Dr. Erik Traneus also contributed to the study design and provided feedback during the final manuscript review and revision stages.

## CONFLICT OF INTEREST STATEMENT

All authors are full‐time employed by RaySearch Laboratories AB where Kjell Eriksson is also a shareholder.

## Supporting information



Supporting Information

## Data Availability

Research data are available upon request in an anonymous format.
