# Supplementary material for: Beyond a constant proton relative biological effectiveness: A survey of clinical and research perspectives among proton institutions in Europe and the United States
Source: J Appl Clin Med Phys. 2024 Nov 3;26(1):e14535. doi: 10.1002/acm2.14535 (PMC11712586; doi:10.1002/acm2.14535)
Supplement: Supplementary file 1 — Supporting Information [file ACM2-26-e14535-s001.pdf]

Proton optimization and evaluation beyond a constant RBE

Introduction to online questionnaire

This online questionnaire is centered around the exploration of optimization and evaluation functions beyond a constant relative biological effectiveness (RBE) in proton therapy. It commences with a section dedicated to general information, followed by the primary survey consisting of a total of 25 questions, which are categorized into clinical utilization, research and experimental endeavors, and future applications related to practices beyond a constant proton RBE. The estimated completion time for the survey is approximately 30 minutes, with responses being saved continuously to allow respondents the flexibility to pause and resume at their convenience. Additionally, a list of abbreviations is available for reference.

The research and experimental activities section specifically focuses on functionalities beyond a constant RBE within research versions of the RayStation treatment planning system (TPS). However, the questionnaire aims to gather comprehensive insights on the topics, and participants are encouraged to provide additional details on their usage in the free-text field for comments, which is available after every question.

Proton optimization and evaluation beyond a constant RBE

Abbreviations and explanations

**CSI**	Craniospinal irradiation
**Dirty dose with LET threshold**	In RayStation the dose can be split into two components depending on if it is delivered with an e.g., LET above a threshold LET or not. The “dirty dose” is then the dose delivered by events above the specified threshold.
**D_{RBE}**	Relative biological effectiveness (RBE)-weighted dose
**$D_{RBE=1.1}$**	D_{RBE} using a constant RBE of 1.1
**DSB model**	RBE model based on the induction of double-strand breaks
**D_{vRBE}**	D_{RBE} using an arbitrary variable RBE model
**H&N**	Head and neck
**LEM**	Local effect model
**LET**	Linear energy transfer
**LET with dose threshold**	Only LET in voxels with a dose higher than the specified dose threshold are considered
**LET_d**	Dose-averaged linear energy transfer
**LET_t**	Track-averaged linear energy transfer
**Linear RBE-LET model**	RBE model as RBE = $a + b \cdot$ LET, where a and b are constants of choice
**MKM**	Microdosimetric-kinetic model
**OAR**	Organ at risk
**RBE**	Relative biological effectiveness
**RMF**	Repair-misrepair-fixation
**RTT**	Radiotherapy technologist
**TPS**	Treatment planning system

Proton optimization and evaluation beyond a constant RBE

General information

What is your profession(s)? (multiple choice)

☐ Physicist

☐ Physician

☐ Dosimetrist/RTT

☐ Doctoral candidate

☐ Researcher

☐ Other

Comments:

How many years have you used RayStation?

○ 1-3 years ○ 4-6 years ○ 7-9 years ○ 10+ years

Comments:

Which research version(s) of RayStation have you been using? (multiple choice)

☐ 6 (5.99.50) ☐ 7 (6.99.50) ☐ 9A (8.99.30) ☐ 10B (10.1.130) ☐ 11B (12.0.130)

Comments:

Proton optimization and evaluation beyond a constant RBE

Clinical use (1/2)

1. Which site(s) do you treat with protons at your institution? (multiple choice)

☐ All treatment sites ☐ Breast ☐ CSI

☐ Eye ☐ Gynecological cancers ☐ Gastrointestinal cancers

☐ H&N ☐ Intracranial lesions ☐ Liver

☐ Lung ☐ Lymphoma ☐ Sarcoma

☐ Skin cancers ☐ Urogenital cancers ☐ Other sites

Comments:

2. Do you prescribe dose, optimize, or evaluate proton treatment plans using any other RBE model than the constant RBE of 1.1?

○ Yes ○ No

3a. If 'Yes' on Q2: Which RBE model(s) do you use? (multiple choice)

☐ Carabe et al. 2012 ☐ McNamara et al. 2015 ☐ Wedenberg et al. 2013

☐ LEM ☐ MKM ☐ RMF model

☐ Linear RBE-LET model ☐ DSB model ☐ Other model(s)

3b. If 'No' on Q2: Which option(s) describe your clinical practice? (multiple choice)

☐ We are only interested in RBE=1.1 for proton therapy

☐ We would like to use variable RBE models if we had the tools, clinical data etc.

☐ We use RBE=1.1, but mitigate potential variable RBE in OARs using other strategies

☐ We use RBE=1.1, but exploit potential variable RBE in tumors using other strategies

☐ None of the above

Comments:

Proton optimization and evaluation beyond a constant RBE

Clinical use (2/2)

4. Where do you actively consider a potential variable RBE? (multiple choice)

☐ All OARs ☐ Selected OARs ☐ Tumors ☐ Nowhere

Comments:

5. What strategies do you use to mitigate/exploit a potentially variable RBE in plan optimization? (multiple choice)

☐ Avoid beams/spots stopping in OARs
☐ Use special beam arrangement(s)
☐ Reduce target dose adjacent to OARs
☐ Increase # of Bragg peaks in the tumor
☐ No specific strategy
☐ Other strategy
☐ Avoid Bragg peaks close to the surface
☐ Robust optimization
☐ Use stricter clinical goals for OARs
☐ Use LET-based optimization functions
☐ Use variable RBE functions in optimization

Comments:

6. What strategies do you use to mitigate/exploit a potentially variable RBE in plan evaluation? (multiple choice)

☐ Evaluating LET distributions
☐ Evaluating D_{RBE} distributions using variable RBE model(s)
☐ Evaluating proton track-end distributions
☐ Evaluating beam angles, spot map and Bragg peaks positions
☐ No evaluations beyond RBE=1.1
☐ Other strategy

Comments:

Proton optimization and evaluation beyond a constant RBE

Research and experimental activities (1/6)

Based on your experience with RayStation, please evaluate the functions beyond a constant RBE for plan optimization (Q7-9) and plan evaluation (Q10-12) below.

7. Which of the following functions have you used in plan optimization?

Function	Used	Not used	Not available
Variable RBE model	◯	◯	◯
Linear RBE-LET model	◯	◯	◯
Dirty dose with LET threshold	◯	◯	◯
LET_d with dose threshold	◯	◯	◯
Proton track-ends	◯	◯	◯

8. The functions you use in plan optimization, what are they used for? (if 'Used' in Q7)

Function	OARs	Targets	Both
Variable RBE model	◯	◯	◯
Linear RBE-LET model	◯	◯	◯
Dirty dose with LET threshold	◯	◯	◯
LET_d with dose threshold	◯	◯	◯
Proton track-ends	◯	◯	◯

Proton optimization and evaluation beyond a constant RBE

Research and experimental activities (2/6)

9. How useful would you grade the plan optimization functions you use at your institution? (if ‘Used’ in Q7)

Function	Not useful				Very useful	No opinion
Variable RBE model	○ 1	○ 2	○ 3	○ 4	○ 5	○
Linear RBE-LET model	○ 1	○ 2	○ 3	○ 4	○ 5	○
Dirty dose with LET threshold	○ 1	○ 2	○ 3	○ 4	○ 5	○
LET_d with dose threshold	○ 1	○ 2	○ 3	○ 4	○ 5	○
Proton track-ends	○ 1	○ 2	○ 3	○ 4	○ 5	○

Comments:

Proton optimization and evaluation beyond a constant RBE

Research and experimental activities (4/6)

10. Which of the following functions have you used in plan evaluation?

Function	Used	Not used	Not available
Variable RBE model	○	○	○
Linear RBE-LET model	○	○	○
Dirty dose with LET threshold	○	○	○
LET_d with dose threshold	○	○	○
LET_t with dose threshold	○	○	○
Proton track-ends	○	○	○

11. The functions you use in plan evaluation, what are they used for? (if 'Used' from Q10)

Function	OARs	Targets	Both
Variable RBE model	○	○	○
Linear RBE-LET model	○	○	○
Dirty dose with LET threshold	○	○	○
LET_d with dose threshold	○	○	○
LET_t with dose threshold	○	○	○
Proton track-ends	○	○	○

Proton optimization and evaluation beyond a constant RBE

Research and experimental activities (4/6)

12. How useful would you grade the plan evaluation functions you use at your institution? (if ‘Used’ from Q10)

Function	Not useful			Very useful		No opinion
Variable RBE model	◯ 1	◯ 2	◯ 3	◯ 4	◯ 5	◯
Linear RBE-LET model	◯ 1	◯ 2	◯ 3	◯ 4	◯ 5	◯
Dirty dose with LET threshold	◯ 1	◯ 2	◯ 3	◯ 4	◯ 5	◯
LET_d with dose threshold	◯ 1	◯ 2	◯ 3	◯ 4	◯ 5	◯
LET_t with dose threshold	◯ 1	◯ 2	◯ 3	◯ 4	◯ 5	◯
Proton track-ends	◯ 1	◯ 2	◯ 3	◯ 4	◯ 5	◯

Comments:

Proton optimization and evaluation beyond a constant RBE

Research and experimental activities (5/6)

13. Have you used other special functionalities in the research versions of RayStation such as alanine detector response, energy spectrum per voxel etc.? (free-text answer)

Answer:

14. For which type of treatment courses have you explored the use of optimization functions beyond RBE=1.1? (multiple choice)

☐ Primary treatment courses

☐ Re-irradiation courses

Comments:

15. For which type of tissues have you explored the use of optimization functions beyond RBE=1.1? (multiple choice)

☐ Parallel structured OARs

☐ Serial structured OARs

☐ Grade I-II toxicities

☐ Grade III-IV toxicities

☐ Low α/β tumors (<5 Gy)

☐ Medium high α/β tumors (5-15 Gy)

☐ High α/β tumors (>15 Gy)

☐ Other

Comments:

Proton optimization and evaluation beyond a constant RBE

Research and experimental activities (6/6)

16. Do you alter the beam arrangement compared to the clinical one when adding optimization functions beyond RBE=1.1?

◌ Yes ◌ No

Comments:

17. If ‘Yes’ on Q16: Which option(s) fit your strategy? (multiple choice)

☐ Avoid beams/spots stopping in the OARs of most interest

☐ Add extra beam(s) to clinical beam(s)

☐ Alter clinical beam angle(s) in other ways

☐ Other special beam arrangements

☐ None of the above

Comments:

18. How many beams do you typically use? If multiple answers, please comment on the beam selection. (multiple choice)

☐ 1-2 ☐ 2-3 ☐ 3-4 ☐ 4 or more

Comments:

Proton optimization and evaluation beyond a constant RBE

Future use (1/4)

19. Which treatment sites do you think would benefit from optimization and/or evaluation beyond dose? (multiple choice)

☐ All treatment sites	☐ Breast	☐ CSI
☐ Eye	☐ Gynecological cancers	☐ Gastrointestinal cancers
☐ H&N	☐ Intracranial lesions	☐ Liver
☐ Lung	☐ Lymphoma	☐ Sarcoma
☐ Skin cancers	☐ Urogenital cancers	☐ Other sites

Comments:

20. Which default linear energy transfer (LET) method do you prefer for evaluation and optimization in a clinical treatment planning system when it comes to…

(a) …the LET averaging method?

○ Dose-averaged ○ Track-averaged ○ No averaging ○ Other

(b) …the medium where LET is computed?

○ Local medium ○ Unit density medium ○ Unit density water ○ Other

(c) …the particles included in the LET computation?

○ Primary protons ○ All protons ○ All ions ○ Other

Comments:

Proton optimization and evaluation beyond a constant RBE

Future use (2/4)

21. Which proton RBE model(s) would you prefer to be available in a clinical TPS? (multiple choice)

☐ Carabe et al. 2012 ☐ McNamara et al. 2015 ☐ Wedenberg et al. 2013

☐ LEM ☐ MKM ☐ RMF model

☐ Linear RBE-LET model ☐ DSB model ☐ Only RBE=1.1

☐ Other model(s)

Comments:

22. If functions beyond RBE=1.1 were clinically available for plan optimization, how do you intend to use them? (multiple choice)

☐ Routinely for every patient ☐ For specific cases

☐ TCP using variable RBE ☐ NTCP using variable RBE

☐ No clinical use for variable RBE models ☐ Other ways

Comments:

Proton optimization and evaluation beyond a constant RBE

Future use (3/4)

23. If functions beyond RBE=1.1 were clinically available for plan evaluation, how do you intend to use them? (multiple choice)

☐ Routinely for every patient

☐ For specific cases

☐ TCP using variable RBE

☐ NTCP using variable RBE

☐ No clinical use for variable RBE models

☐ Other ways

Comments:

24. Would you be interested in participating in a planning study addressing the use of optimization functions beyond dose for proton therapy in RayStation?

○ Yes ○ No

Comments:

Proton optimization and evaluation beyond a constant RBE

Future use (4/4)

25. Are we allowed to use your feedback for marketing purposes, knowledge sharing, journal publications, conference abstracts etc.?

○ Yes ○ Yes, but anonymously ○ No

Comments:
